# The Impact of an Integrated County–Township–Village Care Model on Type 2 Diabetes Management in Resource-Constrained Areas in China: A Synthetic Control Method

**DOI:** 10.3390/healthcare14203450

**Published:** 2026-10-15

**Authors:** Jingbo Wang, Xing Xing, Shimeng Liu, Yan Wei, Yingyao Chen

**Affiliations:** 1 School of Public Health, Fudan University, Shanghai 200032, China; 16111020033@fudan.edu.cn (J.W.);; 2 Center for Project Supervision and Management, National Health Commission, Beijing 100044, China; 3 School of Public Health, Peking University, Beijing 100191, China; xx@bjmu.edu.cn; 4 National Health Commission Key Laboratory of Health Technology Assessment, Fudan University, Shanghai 200032, China

**Keywords:** type 2 diabetes mellitus, integrated care, glycemic control, primary health care, synthetic control method

## Abstract

**Objective:** The objective of this study is to evaluate county-specific changes in standardized management and glycemic control associated with an integrated county–township–village care model among patients with type 2 diabetes mellitus (T2DM) in resource-constrained areas of China. **Methods:** Three intervention counties that implemented an integrated county–township–village care model in 2019 were included. County-level quarterly data from 2017 to 2022 were extracted from electronic health record systems. Two outcome measures were assessed: the standardized management rate and the glycemic control rate among patients with T2DM. Synthetic controls were constructed from non-intervention counties within the same prefecture-level city. Sensitivity analyses excluding the highest-weight donor were conducted. **Results:** The synthetic controls approximated pre-intervention characteristics and outcome trajectories, although fit varied across counties and outcomes. Following implementation, mean standardized management rates exceeded their synthetic counterparts by 4.57, 3.96, and 4.35 percentage points in Counties Q, Y, and F, respectively. Placebo tests provided suggestive evidence for Counties Q and Y (*p* = 0.100 and *p* = 0.111) but limited evidence for County F (*p* = 0.750). Mean glycemic control rates exceeded their synthetic counterparts by 4.61 and 3.81 percentage points in Counties Q and F, respectively, compared with 0.83 percentage points in County Y. Estimated differences remained positive after excluding the highest-weight donor, although their magnitudes were sensitive to donor selection in some models. **Conclusions:** The integrated county–township–village care model was associated with improvements in selected T2DM management outcomes, with patterns varying across counties. Our findings suggest potential value in strengthening collaboration between county hospitals and primary health care institutions in comparable resource-constrained settings.

## 1. Introduction

The prevalence of type 2 diabetes mellitus (T2DM) is increasing worldwide, accompanied by a substantial burden of diabetes-related complications and associated economic costs for both private and public finances [1,2]. This trend is particularly pronounced in China, where aging, lifestyle changes, and urbanization have contributed to a marked increase in diabetes prevalence [3]. In 2021, an estimated 140.9 million adults aged 20–79 years in China had diabetes, up from 90 million in 2011, accounting for approximately one-quarter of the global diabetes population in this age group [4]. National surveillance data from 2018–2019 indicate that the prevalence of diabetes among Chinese adults aged ≥ 18 years was 12.4%, and more than 90% of all diabetes cases in China are T2DM [5]. T2DM is associated with a wide range of long-term complications, including cardiovascular disease (CVD), cerebrovascular disease, diabetic nephropathy, and diabetic retinopathy, which contribute substantially to disability and premature mortality [2,5]. Notably, CVD was the leading cause of death, accounting for more than 45% of all deaths among the general population in both urban and rural China in 2020 [6], and it is also a major complication and cause of death among patients with diabetes, underscoring the need for comprehensive management of diabetes and its associated risk factors for complications [2,5,7]. Therefore, strengthening long-term management of T2DM and improving glycemic control are essential for reducing complications and mitigating the growing disease burden.

In response to the growing burden of diabetes, China has incorporated diabetes prevention and control into its national health strategy [3]. Since 2009, the National Basic Public Health Services Program (NBPHSP) has provided free T2DM management services for residents aged ≥ 35 years with T2DM through primary health care (PHC) institutions, including at least four face-to-face follow-up visits per year, lifestyle counseling, medication monitoring, and annual health examinations [8]. During 2009–2019, substantial progress was achieved, with notable improvements in the proportion of patients receiving standardized management and achieving glycemic control. One study reported that the glycemic control rate (based on fasting blood glucose < 7.0 mmol/L at the most recent follow-up) among T2DM patients managed under NBPHSP increased from 38.86% in 2009 to 63.55% in 2019, a gain of 24.69 percentage points [9]. However, the overall level of glycemic control among Chinese patients remains suboptimal [7,10]. For example, national survey data using a different measure of glycemic control showed that only 50.1% of adults receiving treatment for diabetes achieved glycated hemoglobin (HbA1c) < 7.0% in 2018 [7]. In addition, limitations in PHC workforce capacity, insufficient coordination between PHC institutions and hospitals, and gaps in continuity of care pose challenges to effective chronic disease management [11], highlighting the need to strengthen coordinated diabetes care in resource-constrained settings.

Despite considerable efforts to expand the supply of diabetes management services, delivering standardized services and maintaining glycemic control remain challenging, particularly in resource-constrained areas. Strengthening integrated and continuous care across different levels of the healthcare system may therefore improve diabetes management outcomes [12,13]. Previous studies have demonstrated that community health worker interventions can modestly improve glycemic control [14], while better medication and dietary adherence is associated with a higher likelihood of achieving glycemic control among community-managed patients with T2DM [15]. A hospital-based cross-sectional study also found that higher implementation fidelity of comprehensive geriatric assessment was associated with lower HbA1c levels in older adults with T2DM [16]. Nevertheless, previous studies have primarily examined individual- or community-level interventions [14,15,16], whereas evidence on the effectiveness of integrated diabetes management at the broader county health-system level, involving coordination across county hospitals, township health centers, and village clinics, remains limited in resource-constrained areas.

To address this gap, this study aimed to evaluate county-specific changes in T2DM management outcomes associated with the implementation of an integrated county–township–village care model in three resource-constrained counties in China, using the synthetic control method. These findings may provide evidence to support future scale-up of this integrated intervention model and to inform efforts to strengthen coordination between hospitals and PHC institutions and improve T2DM management in resource-constrained areas.

## 2. Methods

### 2.1. Study Design and Study Settings

The intervention used existing local health resources to develop an integrated county–township–village care model based on PHC institutions and supported by county hospitals [17,18]. This model established multidisciplinary care teams comprising county hospital specialists, township health center general practitioners, public health professionals, and village doctors and emphasized a comprehensive approach to provide continuous services aligned with existing national health policies. The core components included (1) encouraging PHC institutions to identify and enroll patients through routine clinical services and health examinations; (2) conducting comprehensive health examinations for enrolled patients by assessing health status, risk factors, and medication use; (3) developing individualized care plans based on examination results; (4) delivering integrated hospital-based treatment and community-based long-term care according to patients’ health status, with clinical specialists from county-level hospitals and general practitioners from PHC institutions providing medical examinations, medication guidance, and referrals and public health workers and village doctors supporting lifestyle interventions; (5) implementing risk-stratified follow-up on a monthly or quarterly basis, using standardized national criteria to assess disease control, and providing self-management support and medication adherence counseling; and (6) conducting annual health examinations and reassessments to dynamically adjust subsequent management plans.

The intervention formed part of a pilot project targeting economically disadvantaged counties in central and western China with PHC capacity at an intermediate level relative to other counties in their respective provinces. Pilot counties were selected non-randomly through recommendations from provincial authorities and review by the central project office. Selection criteria included (1) the willingness of local health authorities and relevant departments to participate in and implement innovative care models and provide managerial support; (2) relatively low levels of economic development; (3) the capacity to deliver basic public health services and the presence of a village clinic in every village; (4) an established county-level electronic health record (EHR) system; and (5) the capacity of county-level centers for disease control and prevention (CDCs) to provide technical guidance and monitoring. Ultimately, thirteen counties were selected to participate in the intervention program. The pilot counties were selected in the same batch in 2017. The project progress reports indicated that 2017–2018 was the period preceding full implementation, during which the pilot counties primarily undertook preparatory activities, including interdepartmental coordination, technical training, public awareness campaigns, and information system development.

Three intervention counties were selected from these sites based on the following criteria: (1) a common quarter of full implementation; (2) continued intervention delivery thereafter; (3) availability of quarterly county-level monitoring data for 2017–2022; and (4) willingness to provide the required data. Implementation timing and continuity were assessed using the central project plan and progress reports, together with county-specific implementation schedules and work reports. Finally, three counties were included: County Q in Province S, County Y in Province N, and County F in Province B. These counties shared resource constraints but also met the organizational and service-capacity requirements for participation in the pilot project. Full implementation across all PHC institutions, including village clinics, began in the first quarter of 2019 in all three counties. This quarter was therefore designated as the onset of full implementation for the analysis. The pre-intervention period comprised eight quarters from January 2017 to December 2018, and the post-intervention period comprised 16 quarters from January 2019 to December 2022. Quarterly county-level observations were used for all analyses.

A separate donor pool was defined for each intervention county using non-intervention counties within the same prefecture-level city. Donor counties were required to (1) be outside the pilot intervention program; (2) have implemented no similar interventions during the study period; (3) have comparable healthcare policies, insurance schemes, and PHC service arrangements for T2DM management; and (4) have complete data required for the analyses. Restricting donor selection to the same prefecture-level city was intended to reduce differences in regional context and policy implementation. The donor pools comprised nine counties for County Q, eight for County Y, and seven for County F.

### 2.2. Data Sources

County-level socioeconomic and health system characteristics were obtained from county statistical reports (2017–2022) and data provided directly by the participating counties. Data on quarterly T2DM management outcomes during 2017–2022 were extracted from county EHR systems, with reporting dates corresponding to the last day of each calendar quarter. The EHR systems maintained longitudinal records of demographic characteristics, health examinations, and chronic disease management services delivered by PHC institutions under the NBPHSP, including follow-up visits and medication guidance records. These records were collected for routine PHC service delivery and monitoring. In this study, outcome data were aggregated at the county level for each quarter, and the unit of analysis was the county-quarter.

Multiple quality-control procedures were implemented at the PHC institution, county, and provincial levels to support the accuracy, completeness, and consistency of the data. At the PHC institution level, healthcare providers were responsible for verifying the completeness and accuracy of records during data entry, supported by automated logic and consistency checks embedded within the EHR systems. At the county level, health departments conducted regular quality checks of EHRs, including verification of randomly selected source records, outlier detection, and patient surveys to verify recorded service delivery. At the provincial level, provincial authorities routinely monitored NBPHSP data quality and provided guidance.

In this study, additional quality-control procedures were undertaken before analysis. Uniform variable definitions and coding rules were applied across all study counties, including intervention and donor counties, and consistency checks were performed to assess the comparability of study variables and outcome measures. However, these procedures could not establish whether glucose measurement practices were consistent across counties or over time. County-level data on glucose-testing frequency and timing, and on the proportions of fasting and non-fasting measurements were unavailable, preventing direct assessment of whether these practices changed following intervention implementation.

This study retrospectively analyzed routinely collected, de-identified, county-level aggregated data to evaluate an existing intervention. It involved no additional study-administered intervention or biological sample collection. Ethical approval was obtained from the Peking University Institutional Review Board (No. IRB00001052-22155).

### 2.3. Study Variables

**Outcomes**: The county-level standardized management rate among patients with T2DM was used to reflect the proportion of managed patients receiving services that met the specified management requirements. The county-level glycemic control rate among patients with T2DM was used to reflect the proportion of managed patients with recorded follow-up fasting blood glucose meeting the control target, rather than overall health status or individual-level clinical effects. Both indicators were calculated as percentages at the end of each calendar quarter. Detailed definitions, denominators, and quarterly calculation rules are provided in Table 1.

**Intervention status**: The primary exposure was based on whether a county had fully implemented the intervention. The first quarter of 2019, when the intervention was fully implemented in all three study counties, was designated as the intervention time point. A time-varying binary indicator was constructed, coded as 0 through the fourth quarter of 2018, 1 from the first quarter of 2019 onward for intervention counties, and 0 throughout the study period for donor counties.

**Predictors**: This study included county-level socioeconomic and health resource characteristics measured in 2017 as predictors for constructing synthetic controls [19]: (1) per capita GDP (10,000 RMB) to reflect economic development; (2) the proportion of county-level annual fiscal health expenditure in total general public budget expenditure (%) and per capita allocation of NBPHSP funds (RMB) to indicate the relative priority given to health in local government spending and NBPHSP funding, respectively; and (3) the numbers of health technicians and hospital beds per 1000 population to represent health resource availability. These variables were selected to characterize economic and health-system conditions potentially related to service delivery and the study outcomes. In addition, the eight quarterly values of the corresponding outcome during 2017–2018 were included as predictors to match pre-intervention outcome trajectories for each county-specific synthetic control.

### 2.4. Data Analysis

The synthetic control method (SCM) was used to estimate county-specific differences in T2DM management outcomes associated with intervention implementation [20,21]. This approach constructs a counterfactual as a weighted combination of outcomes from donor counties, with weights selected to approximate the pre-intervention characteristics and outcome trajectory of each intervention county. Post-intervention differences between observed and synthetic outcomes were interpreted as estimated intervention effects under the assumptions of adequate pre-intervention fit, no intervention spillover to donor counties, and no differential concurrent changes that would invalidate the counterfactual comparison. The SCM does not eliminate all sources of unmeasured confounding. Using the donor pools described above, a separate synthetic control was constructed for each intervention county and for each of the two outcomes, yielding six models. No pooled effect across the three counties was estimated.

The analysis was conducted using the “scm” package in R version 4.2.0. To assess whether the estimated intervention effects were unusual relative to those obtained for non-intervention counties, a placebo test was performed following the synthetic control framework [22].

Following Abadie et al. [21], let county 1 denote the intervention county and counties 2, …, *K* + 1 denote its *K* donor counties. Let *T*_0_ = 8 denote the final pre-intervention quarter, corresponding to the fourth quarter of 2018, and *T* = 24 denote the final observation quarter. The intervention period therefore begins at *t* = *T*_0_ + 1, the first quarter of 2019. The estimated counterfactual outcome was

$${\hat{Y}}_{1t}^{N}=\sum_{j=2}^{K+1}w_{j}Y_{jt},\;w_{j}\geq 0,\;\sum_{j=2}^{K+1}w_{j}=1.$$

Here, *Y_jt_* denotes the observed outcome in donor county *j* at quarter *t*, *N* denotes the absence of the intervention, and *w_j_* denotes the donor weight estimated using pre-intervention data.

The donor weights were estimated using pre-intervention data to minimize discrepancies between the intervention county and its synthetic control in the selected socioeconomic and health resource predictors and the eight quarterly values of the corresponding outcome. The estimated quarterly intervention effect was defined as the observed-minus-synthetic difference:

$${\hat{\tau }}_{1t}=Y_{1t}-{\hat{Y}}_{1t}^{N},\;t=T_{0}+1,...,T.$$

For each county and outcome, the mean post-intervention difference was calculated across the 16 quarters from 2019 through 2022:

$${\bar{\hat{\tau }}}_{1}=\frac{1}{T-T_{0}}\sum_{t=T_{0}+1}^{T}{\hat{\tau }}_{1t}.$$

The final-quarter difference, corresponding to the fourth quarter of 2022, was also reported. Outcomes were analyzed on a 0–1 proportion scale, and estimated differences were multiplied by 100 for presentation in percentage points. Positive differences indicated higher observed rates than those of the synthetic control.

Pre-intervention fit was assessed using covariate balance and the root mean squared prediction error (RMSPE):

$${RMSPE}_{pre}=\sqrt{\frac{1}{T_{0}}\sum_{t=1}^{T_{0}}{\left(Y_{1t}-{\hat{Y}}_{1t}^{N}\right)}^{2}}$$

Post-intervention RMSPE was calculated analogously over the 16 post-intervention quarters. The post-/pre-intervention RMSPE ratio was reported to characterize post-intervention divergence relative to pre-intervention fit. Supplementary Table S3 reports RMSPE ratios, whereas the placebo comparisons in Supplementary Figures S1 and S2 use MSPE ratios, which are the squares of the corresponding RMSPE ratios.

Placebo tests were conducted by assigning a hypothetical intervention beginning in the first quarter of 2019 to each donor county and re-estimating its synthetic control [21,22]. The intervention county’s post-/pre-intervention prediction-error ratio was compared with the corresponding placebo ratios. The estimated placebo effects were then compared with the effect estimated for the intervention county to evaluate their relative magnitude [23]. This approach follows the permutation-based framework used in synthetic control studies. All donor counties were retained. The *p*-value was calculated by adding one to the number of donor counties with MSPE ratios greater than or equal to that of the intervention county and dividing by the number of donor counties plus one. Ties were therefore counted as equally extreme. Therefore, the minimum attainable *p*-values were 0.100, 0.111, and 0.125 for Counties Q, Y, and F, respectively. Given the small donor pools and the resulting limited resolution of these comparisons, placebo-based *p*-values were reported and interpreted alongside effect magnitudes and fit diagnostics. Under this procedure, no attainable *p*-value could meet the conventional 0.05 significance threshold. For example, certain studies set α = 0.20 as an exploratory threshold for detecting potential signals of intervention effects under small-sample conditions [24,25]. These applications provide empirical precedent rather than a general methodological justification for this threshold. Findings meeting this threshold were interpreted as suggestive rather than confirmatory evidence.

Sensitivity to donor selection was assessed by excluding the maximum-weight donor from each of the six models and re-estimating the synthetic control using the remaining donors [21]. The resulting mean post-intervention differences and pre-intervention RMSPE values were compared with those from the original models. Relative changes in mean post-intervention differences were calculated by subtracting the original estimate from the re-estimated value, dividing by the original estimate, and multiplying by 100 to express the change as a percentage. Changes in fit were summarized as the ratio of re-estimated-to-original pre-intervention RMSPE.

## 3. Results

### 3.1. Construction of Synthetic Control Groups

The synthetic control models were assessed using pre-intervention covariate balance and outcome fit for both the standardized management rate and the glycemic control rate among patients with T2DM. Across all three intervention counties (Q, Y, and F), the absolute values of the pre-intervention relative differences in the selected socioeconomic and health resource predictors were generally smaller between intervention counties and their synthetic controls than between intervention counties and the corresponding donor-pool means. For both outcomes, the absolute values of these relative differences were smaller for synthetic controls than for donor-pool means in all eight pre-intervention quarters in each county (Table 2 and Table 3).

Predictor weight distributions varied by county and outcome specification (Supplementary Table S1). The eight pre-intervention quarterly outcome values collectively accounted for most of the predictor weight in all six models, with combined weights ranging from 0.606 to 0.877. Among socioeconomic and health resource predictors, the largest weights for the standardized management rate were assigned to per capita basic public health service funding in County Q (0.114), the fiscal health expenditure share in County Y (0.083), and health technician density in County F (0.093). For the glycemic control rate, the corresponding predictors were health technician density in County Q (0.059), per capita GDP in County Y (0.226), and hospital bed density in County F (0.310).

Most donor weight was concentrated in a small number of counties (Supplementary Table S2). For the standardized management rate, donor counties 2 and 9 accounted for 88.7% of the weight in the synthetic control for County Q; counties 7 and 8 accounted for 97.0% for County Y; and county 3 accounted for 80.9% for County F. For the glycemic control rate, the corresponding contributions were 94.1% from counties 3 and 4 for County Q, 100% from counties 6 and 7 for County Y, and 81.4% from counties 3 and 4 for County F.

Pre-intervention RMSPE values for the standardized management rate were 0.00158, 0.00793, and 0.01441 in Counties Q, Y, and F, respectively. Corresponding values for the glycemic control rate were 0.00498, 0.01924, and 0.00479 (Supplementary Table S3). All RMSPE values were calculated on the 0–1 proportion scale.

### 3.2. Standardized Management Rate Following Intervention Implementation

**Figure 1:**
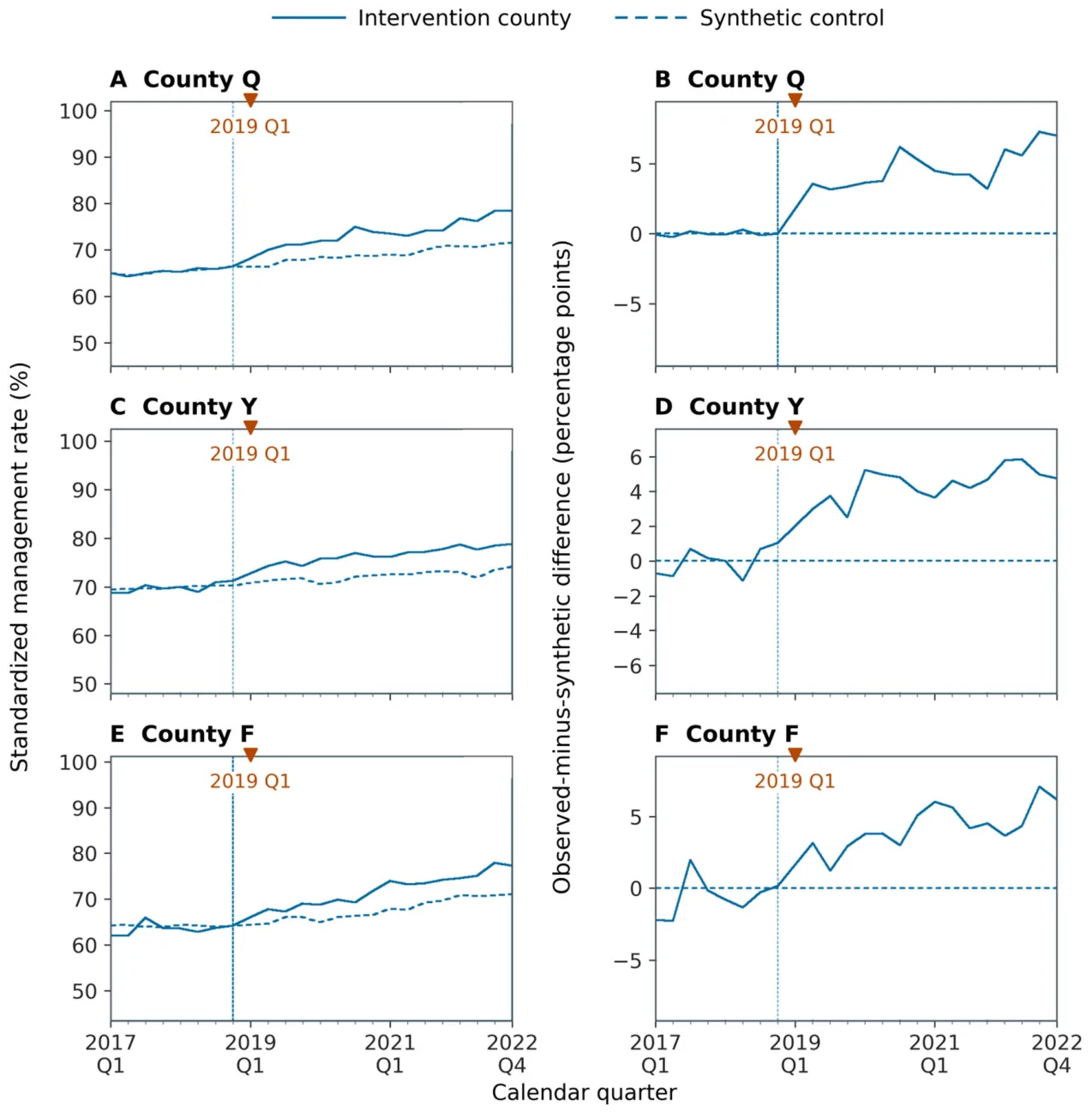
Standardized management rates among patients with T2DM in intervention counties and synthetic controls, 2017–2022. (**A**) Observed and synthetic control rates in County Q; (**B**) observed-minus-synthetic differences in County Q; (**C**) observed and synthetic control rates in County Y; (**D**) observed-minus-synthetic differences in County Y; (**E**) observed and synthetic control rates in County F; (**F**) observed-minus-synthetic differences in County F. Notes. In panels (**A**,**C**,**E**), solid lines represent observed rates in intervention counties and dashed lines represent synthetic control rates; rates are expressed as percentages. In panels (**B**,**D**,**F**), differences are expressed in percentage points. Positive differences indicate higher observed rates; horizontal dashed lines indicate zero. Vertical dotted lines mark the end of the pre-intervention period (2018 Q4), and downward triangles mark full implementation in 2019 Q1. Axis ranges vary across panels. T2DM, type 2 diabetes mellitus.

Pre-intervention fit varied across Counties Q, Y, and F (Figure 1; Supplementary Table S3). During the post-intervention period of 2019–2022, the mean observed-minus-synthetic differences in the standardized management rate were 4.57, 3.96, and 4.35 percentage points in Counties Q, Y, and F, respectively. The final-quarter differences in the fourth quarter of 2022 were 7.10, 4.80, and 6.21 percentage points, respectively (Table S3).

Placebo results are presented in Supplementary Figure S1. Counties Q and Y had the largest post-/pre-intervention MSPE ratios in their respective placebo comparisons, with placebo-based *p*-values of 0.100 and 0.111, respectively. The corresponding *p*-value for County F was 0.750. None met the conventional 0.05 significance threshold. The *p*-values for Counties Q and Y were the minimum attainable in their respective comparison sets and were below the exploratory threshold of 0.20. These findings were interpreted as suggestive rather than confirmatory evidence.

### 3.3. Glycemic Control Rate Following Intervention Implementation

**Figure 2:**
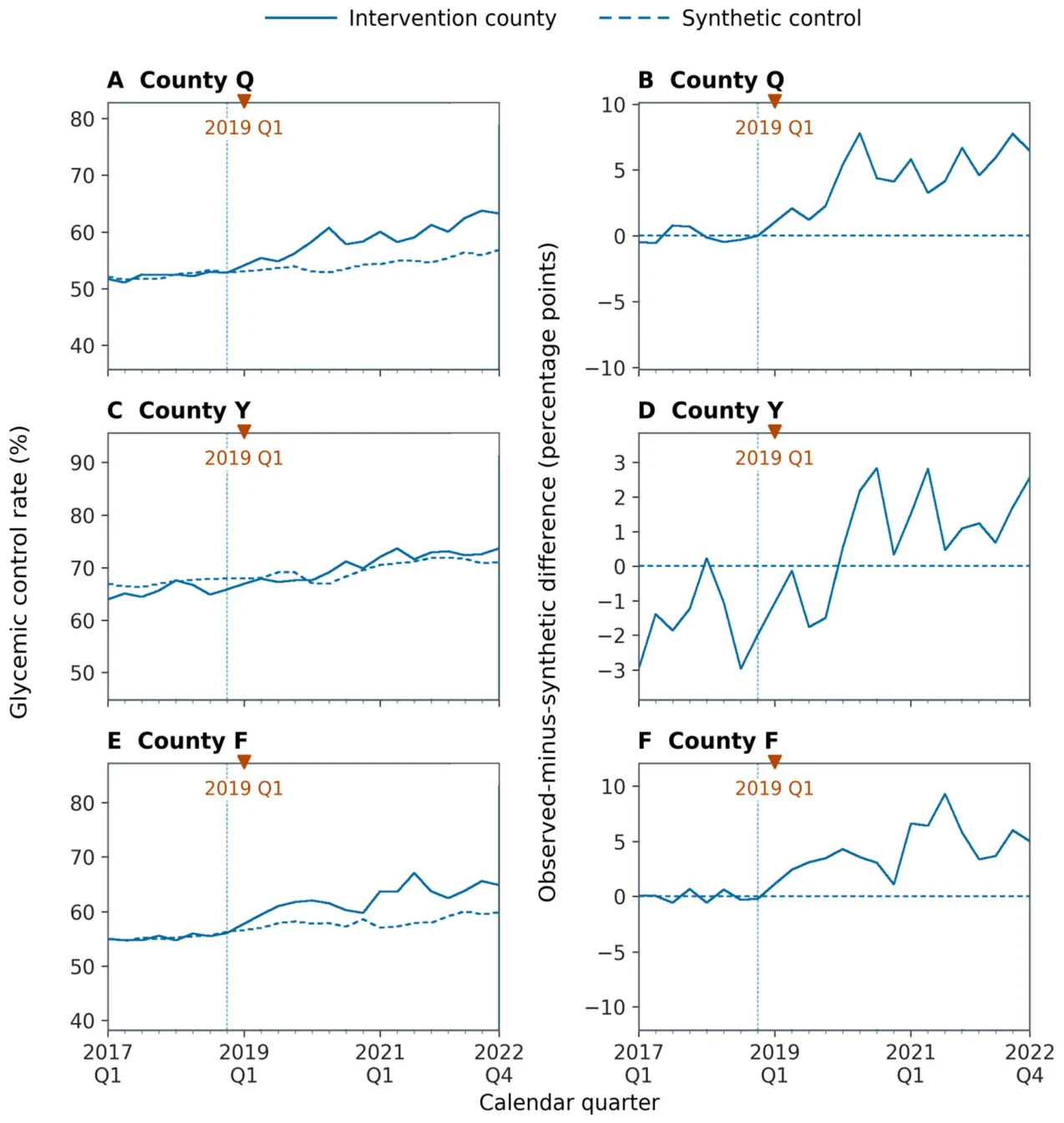
Glycemic control rates among patients with T2DM in intervention counties and synthetic controls, 2017–2022. (**A**) Observed and synthetic control rates in County Q; (**B**) observed-minus-synthetic differences in County Q; (**C**) observed and synthetic control rates in County Y; (**D**) observed-minus-synthetic differences in County Y; (**E**) observed and synthetic control rates in County F; (**F**) observed-minus-synthetic differences in County F. Notes. In panels (**A**,**C**,**E**), solid lines represent observed rates in intervention counties and dashed lines represent synthetic control rates; rates are expressed as percentages. In panels (**B**,**D**,**F**), differences are expressed in percentage points. Positive differences indicate higher observed rates; horizontal dashed lines indicate zero. Vertical dotted lines mark the end of the pre-intervention period (2018 Q4), and downward triangles mark full implementation in 2019 Q1. Axis ranges vary across panels. T2DM, type 2 diabetes mellitus.

Pre-intervention RMSPE was higher in County Y than in Counties Q and F (Figure 2; Supplementary Table S3). During the post-intervention period of 2019–2022, the mean observed-minus-synthetic differences in the glycemic control rate were 4.61, 0.83, and 3.81 percentage points in Counties Q, Y, and F, respectively. The final-quarter differences in the fourth quarter of 2022 were 6.50, 2.50, and 5.12 percentage points, respectively (Table S3).

In-space placebo results are presented in Supplementary Figure S2. Counties Q and F had the largest post-/pre-intervention MSPE ratios in their respective placebo comparisons, with placebo-based *p*-values of 0.100 and 0.125, respectively. The corresponding *p*-value for County Y was 0.889. None met the conventional 0.05 significance threshold. The *p*-values for Counties Q and F were the minimum attainable in their respective comparison sets and were below the exploratory threshold of 0.20. These findings were interpreted as suggestive rather than confirmatory evidence.

### 3.4. Sensitivity Analysis

After excluding the maximum-weight donor from each of the six models, pre-intervention RMSPE increased to 1.07–2.78 times the corresponding original value, with the largest relative increase observed for the standardized management rate in County Q (Supplementary Table S4). The mean post-intervention differences remained positive in all six models.

## 4. Discussion

This study provides suggestive evidence that the implementation of an integrated county-township-village care model was associated with improvements in standardized management and glycemic control among patients with T2DM, with patterns varying across three resource-constrained counties. SCM results showed higher mean post-intervention standardized management rates than those of the synthetic controls in Counties Q and Y, supported by suggestive evidence from placebo tests. Higher mean post-intervention glycemic control rates were also observed in Counties Q and F, with suggestive evidence from the placebo tests. County Y showed a smaller mean difference in glycemic control despite gains in standardized management, while placebo-based evidence for improved standardized management in County F was limited.

The integrated county-level intervention was associated with improvements in standardized T2DM management and glycemic control in some counties. These findings broadly align with prior evidence indicating that integrated care models and quality improvement strategies can enhance selected care processes and clinical outcomes, although benefits vary across interventions and outcomes [26,27]. For example, the SMARTDiabetes trial in China found that a multifaceted digital health intervention combining clinical decision support and patient self-management support reduced HbA1c and fasting plasma glucose levels compared with usual care [28]. Potential mechanisms underlying the observed county-specific improvements may involve the combined influence of strengthened engagement of county-level hospitals, improved coordination across county, township, and village levels, and support for the technical capacity of primary care providers. In particular, iterative cycles of assessment, intervention, and re-evaluation, together with risk-stratified follow-up intensity and structured self-management support, may have supported continuity of care, patient engagement, and adherence to treatment and lifestyle recommendations. These proposed pathways are consistent with the chronic care model and the WHO Innovative Care for Chronic Conditions framework, which emphasize coordinated care, prepared healthcare teams, and self-management support [29,30]. In addition, the COVID-19 pandemic overlapped with the post-intervention period and may have influenced T2DM management. Project records indicated that intervention and donor counties generally followed common provincial and municipal pandemic-response requirements and coordinated health-resource allocation arrangements. Selecting donor counties within the same prefecture-level city may therefore have reduced differences in pandemic-related policies, although differences in local disruptions and service delivery could not be excluded. The intervention continued during the pandemic, and retaining the full 2017–2022 observation period allowed assessment of outcomes during an implementation period that included pandemic-related disruptions. However, the analysis did not isolate pandemic-related effects or directly assess the model’s adaptability.

The observed heterogeneity in county-specific estimates suggests that outcomes associated with the integrated county-level model may be context-dependent. The Consolidated Framework for Implementation Research highlights the potential importance of intervention characteristics, readiness for implementation, available resources, and implementation processes [31]. In this study, project progress reports and third-party evaluations provided contextual information on implementation differences across counties. County Q was reported to have embedded the intervention into the family physician contract service package, established interoperability between clinical and public health information systems, and encouraged patients to engage in self-management. In addition, among the three counties, County Q reportedly had the highest per-patient subsidy standard for PHC personnel delivering intervention services (standardized management of one patient (one health examination and four face-to-face follow-up visits): 160 yuan in County Q versus 110 yuan in County Y and 122 yuan in County F), and the highest proportion of follow-up visits delivered through integrated outpatient services in 2022 (42.2% in County Q, 28.4% in County Y, and 27.7% in County F). Each county followed the subsidy standards of its respective province. These arrangements may have supported continuity of care and coordination across service levels. County Y showed a positive mean difference in the standardized management rate but a smaller mean difference in the glycemic control rate relative to its synthetic control, suggesting that improvements in recorded management processes did not necessarily translate into comparable changes in recorded glycemic control. Its relatively high baseline glycemic control rate among the three counties may have contributed to this pattern, consistent with evidence that diabetes quality improvement interventions often produce larger HbA1c reductions in trials enrolling patients with poorer baseline glycemic control [26]. However, a ceiling effect cannot be established from these county-level data. More broadly, a narrative review of community-based T2DM care models highlights the relevance of health-system integration, workforce support, and infrastructure to implementation and sustainability [32]. These observations support further investigation of contextual influences rather than establishing why outcomes differed across the three counties.

These findings suggest potential value in further evaluating integrated diabetes care in comparable resource-constrained settings, with implementation adapted to local conditions. The variation across counties supports attention to local service capacity and implementation constraints when planning technical support. The differing patterns in standardized management and glycemic control also highlight the need to monitor both service delivery and glycemic outcomes. County hospital support, information sharing, and individualized management warrant further evaluation as potential contributors to these outcomes. Given the sensitivity of the estimates to donor selection, decisions about wider implementation should also be informed by evidence from additional counties and continued outcome monitoring.

The findings should be interpreted in light of several limitations. First, non-random county selection and unmeasured differences in local policies, implementation, and COVID-19-related service disruptions may have influenced the estimates, limiting attribution of the observed differences solely to the intervention. The study did not include a standardized assessment of implementation fidelity across counties, limiting our ability to examine its relationship with the county-specific outcome estimates. Second, the short pre-intervention period and small donor pools limited counterfactual assessment and placebo-based inference. Sensitivity to maximum-weight donor exclusion also warrants caution in interpreting the estimated magnitudes. Third, changes in EHR recording, glucose testing, and patient composition may have affected recorded rates. Fasting blood glucose also provides less information on longer-term glycemic control than HbA1c. Finally, findings from three intervention counties may have limited generalizability to other settings, and county-level estimates cannot establish individual clinical benefits or reductions in complications. Within these limits, this study provides exploratory county-level evidence on integrated T2DM care in resource-constrained settings. Future studies should include additional comparable counties, longer pre-intervention periods, and standardized patient-level and implementation data to assess sustained outcomes and inform wider implementation.

## 5. Conclusions

This study provides suggestive evidence that an integrated county–township–village care model was associated with improvements in standardized T2DM management and recorded glycemic control in three resource-constrained counties in China. Patterns varied across counties, and comparable gains in recorded glycemic control did not consistently accompany improvements in standardized management. These findings suggest the potential value of strengthening collaboration between county hospitals and primary health care institutions, with implementation adapted to local service capacity. Further evaluation in additional counties, using standardized outcome and implementation measures, is needed to assess the sustainability of observed improvements and inform broader implementation.

## Figures and Tables

**Table 1:** Definitions of the county-level standardized management rate and glycemic control rate among patients with T2DM.

Indicator	Indicator Definition
Standardized management rate among patients with T2DM	Standardized management rate = (Number of T2DM patients whose management services met the specified requirements by the end of the reporting quarter)/(Number of T2DM patients who were under management in the county at any time during the reporting quarter, as of the end of the reporting quarter) × 100%. The standardized management rate was calculated quarterly and assessed cumulatively within each calendar year. For patients already enrolled in management at the beginning of the calendar year, compliance was assessed against the following cumulative requirements: at least one face-to-face follow-up visit by the end of Q1, two by the end of Q2, three by the end of Q3, and four by the end of Q4, with at least one visit in each elapsed quarter. For patients newly enrolled during the calendar year, the cumulative requirement began in the quarter of enrollment. All follow-up visits were required to meet the specified content and service requirements. If follow-up identified complications or poor control of blood pressure/blood glucose, an additional follow-up was required within two weeks, as applicable to the patient’s condition. By the end of Q4, patients were also required to have at least one qualifying health examination record for that calendar year; the examination could have taken place in any quarter after enrollment. Cumulative assessment restarted at the beginning of each calendar year.
Glycemic control rate among patients with T2DM	Glycemic control rate = (Number of T2DM patients whose most recent follow-up fasting blood glucose [FBG] measurement during the reporting quarter met the target)/(Number of T2DM patients who were under management in the county at any time during the reporting quarter, as of the end of the reporting quarter) × 100%. The EHR system determined control status using the recorded measurement type and glucose value. Patients without a fasting glucose measurement record during the reporting quarter, including those lost to follow-up, remained in the denominator and were classified as not meeting the control target. The criterion at follow-up was FBG < 7 mmol/L. Glycemic control was defined using fasting blood glucose measurements, with all managed patients included in the denominator. Non-fasting blood glucose < 10.0 mmol/L was not used as an alternative control criterion.

**Table 2:** Pre-intervention relative differences in predictors for the standardized management rate among patients with T2DM.

Predictor	County Q		County Y		County F	
	Intervention vs. Synthetic (%)	Intervention vs. Donor Mean (%)	Intervention vs. Synthetic (%)	Intervention vs. Donor Mean (%)	Intervention vs. Synthetic (%)	Intervention vs. Donor Mean (%)
**Socioeconomic and health resource predictors**						
GDP per capita (10,000 RMB)	3.57	−75.60	−5.33	−33.07	4.40	4.67
Hospital beds per 1000 population	−0.86	22.89	−4.71	13.18	0.87	1.89
Health technicians per 1000 population	−1.26	−24.68	−0.71	−3.33	−3.14	−56.83
Health expenditure as a share of total general public budget expenditure (%)	4.13	5.99	−3.41	20.37	−3.43	−49.43
Basic public health service funding per capita (RMB)	1.84	3.53	0.12	0.23	0.39	0.95
**Pre-intervention quarterly outcome values**						
2017 Q1	−0.11	−10.61	−1.03	−9.47	−3.56	−10.89
2017 Q2	−0.37	−11.46	−1.27	−10.02	−3.69	−9.95
2017 Q3	0.29	−10.37	0.97	−7.45	3.00	−4.79
2017 Q4	−0.08	−9.90	0.23	−8.30	−0.22	−8.48
2018 Q1	−0.08	−11.73	0.00	−8.33	−1.23	−9.50
2018 Q2	0.44	−10.36	−1.62	−9.99	−2.13	−10.87
2018 Q3	−0.15	−11.32	0.96	−7.40	−0.42	−9.20
2018 Q4	−0.02	−11.12	1.44	−6.99	0.30	−8.61

Notes. All entries are relative differences (%), calculated as (intervention county value—comparison value)/intervention county value × 100. The comparison value is the synthetic control value for “Synthetic” and the donor-pool mean for “Donor mean”. Positive values indicate higher values in the intervention county than in the comparison unit; negative values indicate lower values. Units in predictor labels refer to the original variables. GDP, gross domestic product; RMB, renminbi; Q1–Q4, calendar quarters.

**Table 3:** Pre-intervention relative differences in predictors for the glycemic control rate among patients with T2DM.

Predictor	County Q		County Y		County F	
	Intervention vs. Synthetic (%)	Intervention vs. Donor Mean (%)	Intervention vs. Synthetic (%)	Intervention vs. Donor Mean (%)	Intervention vs. Synthetic (%)	Intervention vs. Donor Mean (%)
**Socioeconomic and health resource predictors**						
GDP per capita (10,000 RMB)	4.29	−75.60	−0.48	−33.07	3.17	4.67
Hospital beds per 1000 population	−3.13	22.89	2.45	13.18	0.87	1.89
Health technicians per 1000 population	−5.05	−24.68	0.71	−3.33	0.00	−56.83
Health expenditure as a share of total general public budget expenditure (%)	−1.73	5.99	1.05	20.37	−3.59	−49.43
Basic public health service funding per capita (RMB)	0.61	3.53	0.17	0.23	0.95	0.95
**Pre-intervention quarterly outcome values**						
2017 Q1	−0.87	−12.90	−4.60	−11.47	0.25	−4.47
2017 Q2	−1.04	−13.90	−2.14	−9.49	0.07	−4.02
2017 Q3	1.52	−11.02	−2.91	−11.03	−0.97	−6.05
2017 Q4	1.34	−10.98	−1.89	−9.16	1.21	−3.79
2018 Q1	−0.25	−12.16	0.34	−5.83	−1.01	−7.13
2018 Q2	−0.90	−12.32	−1.58	−7.59	1.12	−5.82
2018 Q3	−0.51	−11.67	−4.58	−12.11	−0.52	−7.09
2018 Q4	0.04	−11.88	−3.01	−10.85	−0.34	−6.38

Notes. All entries are relative differences (%), calculated as (intervention county value—comparison value)/intervention county value × 100. The comparison value is the synthetic control value for “Synthetic” and the donor-pool mean for “Donor mean”. Positive values indicate higher values in the intervention county than in the comparison unit; negative values indicate lower values. Units in predictor labels refer to the original variables. GDP, gross domestic product; RMB, renminbi; Q1–Q4, calendar quarters.

## Data Availability

Data were obtained from the data provider and are available from the authors with the permission of the data provider, following assessment under the Government Information Disclosure Regulation of the People’s Republic of China according to Article 55, and applicable local provisions.

## Supplementary Materials

The following supporting information can be downloaded at https://www.mdpi.com/article/10.3390/healthcare14203450/s1, Figure S1: In-space placebo comparisons for the standardized management rate among patients with T2DM; Figure S2: In-space placebo comparisons for the glycemic control rate among patients with T2DM; Table S1: Predictor weights used to construct the synthetic controls; Table S2: Donor-county weights in the synthetic controls; Table S3: SCM diagnostics and estimated county-specific differences; Table S4: Sensitivity of SCM estimates to maximum-weight donor exclusion.
