# Supplementary material for: The Impact of an Integrated County–Township–Village Care Model on Type 2 Diabetes Management in Resource-Constrained Areas in China: A Synthetic Control Method
Source: Healthcare (Basel). 2026 Oct 15;14(20):3450. doi: 10.3390/healthcare14203450 (PMC13704070; doi:10.3390/healthcare14203450)
Supplement: Supplementary file 1 [file healthcare-14-03450-s001.zip › healthcare-4497481-supplementary.pdf]

Table S1. Predictor weights used to construct the synthetic controls

Predictor	Synthetic County Q		Synthetic County Y		Synthetic County F	
	Standardized management rate of T2DM patients (%)	Glycemic control rate of T2DM patients (%)	Standardized management rate of T2DM patients (%)	Glycemic control rate of T2DM patients (%)	Standardized management rate of T2DM patients (%)	Glycemic control rate of T2DM patients (%)
GDP per capita (10,000 RMB)	0.037	0.029	0.005	0.226	0.001	0
Hospital beds per 1,000 population	0.103	0.043	0	0.056	0.007	0.310
Health technicians per 1,000 population	0.069	0.059	0	0.036	0.093	0.001
Health expenditure as a share of total general public budget expenditure (%)	0.043	0.003	0.083	0.005	0	0
Basic public health service funding per capita (RMB)	0.114	0.003	0.04	0.071	0.02	0.007
Standardized management rate / Glycemic control rate 2017Q1	0.076	0.173	0.133	0.068	0.055	0.033
Standardized management rate / Glycemic control rate 2017Q2	0.074	0.184	0.032	0.09	0.021	0.091
Standardized management rate / Glycemic control rate 2017Q3	0.091	0.062	0.07	0.036	0.029	0.136
Standardized management rate / Glycemic control rate 2017Q4	0.043	0.014	0.142	0.076	0.048	0.136
Standardized management rate / Glycemic control rate 2018Q1	0.085	0.092	0.139	0.014	0.136	0.137
Standardized management rate / Glycemic control rate 2018Q2	0.085	0.207	0.147	0.068	0.269	0.053
Standardized management rate / Glycemic control rate 2018Q3	0.067	0.058	0.094	0.138	0.161	0.056
Standardized management rate / Glycemic control rate 2018Q4	0.113	0.072	0.117	0.116	0.158	0.041

Note. Entries are dimensionless predictor weights for the corresponding county – outcome model. Weights may not sum exactly to 1 because of rounding.

Table S2. Donor-county weights in the synthetic controls

Outcome	Intervention county	Donor-county weights
Standardized management rate of T2DM patients (%)	Q	county-1 (0.002), county-2 (0.352), county-3 (0), county-4 (0), county-5 (0.001), county-6 (0.110), county-7 (0), county-8 (0.001), county 9 (0.535)
	Y	county-1(0.018), county-2(0.003), county-3(0.002), county-4(0.001), county-5(0.001), county-6(0.005), county-7(0.769), county-8(0.201)
	F	county-1(0), county-2(0), county-3(0.809), county-4(0), county-5(0.190), county-6(0), county-7(0)
Glycemic control rate of T2DM patients (%)	Q	county-1(0), county-2(0), county-3(0.227), county-4(0.714), county-5(0), county-6(0), county-7(0), county-8(0.059), county-9(0)
	Y	county-1(0), county-2(0), county-3(0), county-4(0), county-5(0), county-6(0.714), county-7(0.286), county-8(0)
	F	county-1(0), county-2(0.047),county-3(0.572), county-4 (0.242), county-5(0.136), county-6(0.001), county-7(0.001)

Note. Weights may not sum exactly to 1 because of rounding.

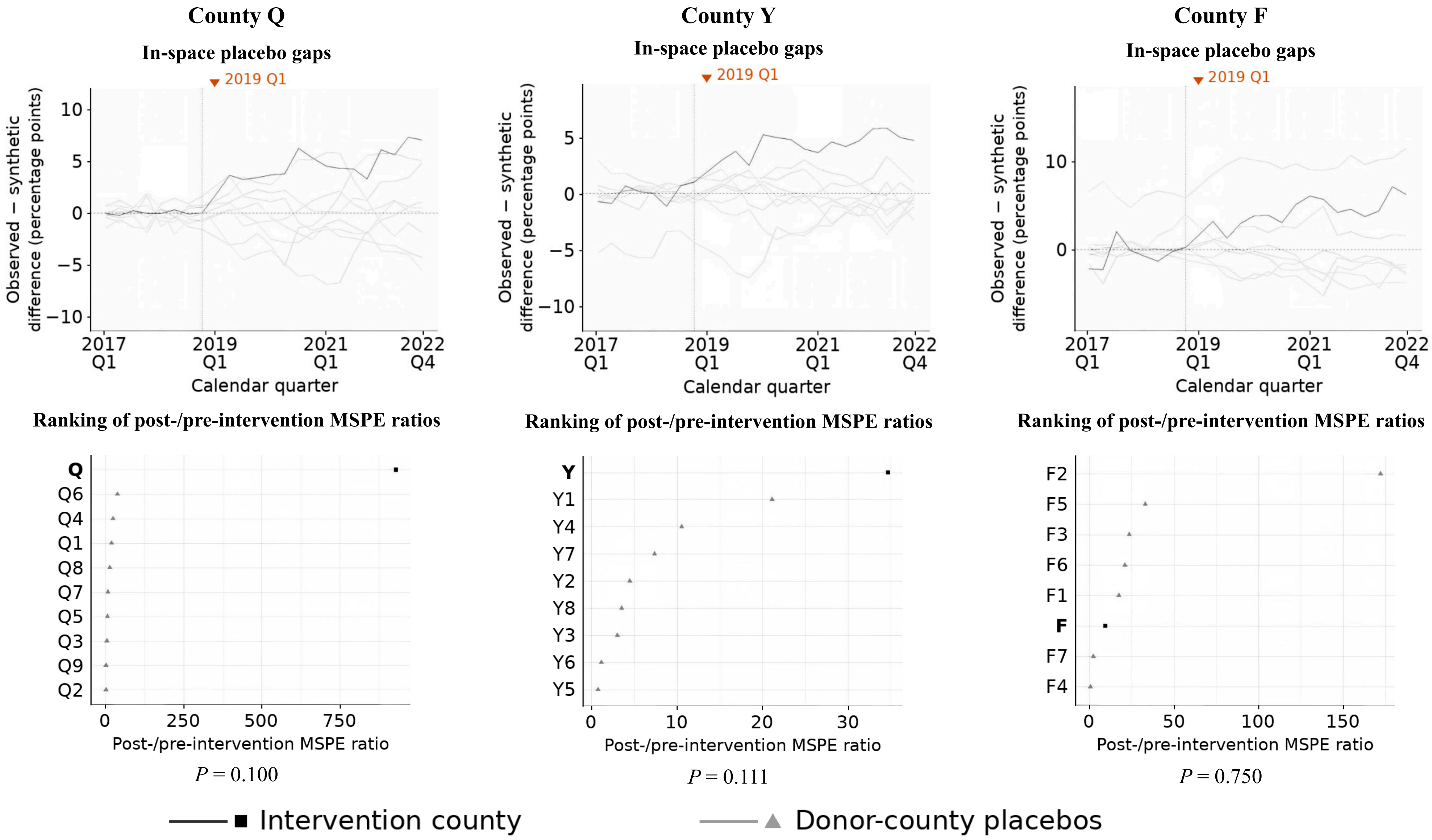

Figure S1. In-space placebo comparisons for the standardized management rate among patients with T2DM.

Notes. Upper panels show quarterly observed-minus-synthetic differences; lower panels rank post-/pre-intervention MSPE ratios. The triangle marks 2019 Q1, the first intervention quarter; the vertical dotted line marks the end of 2018 Q4. MSPE, mean squared prediction error.

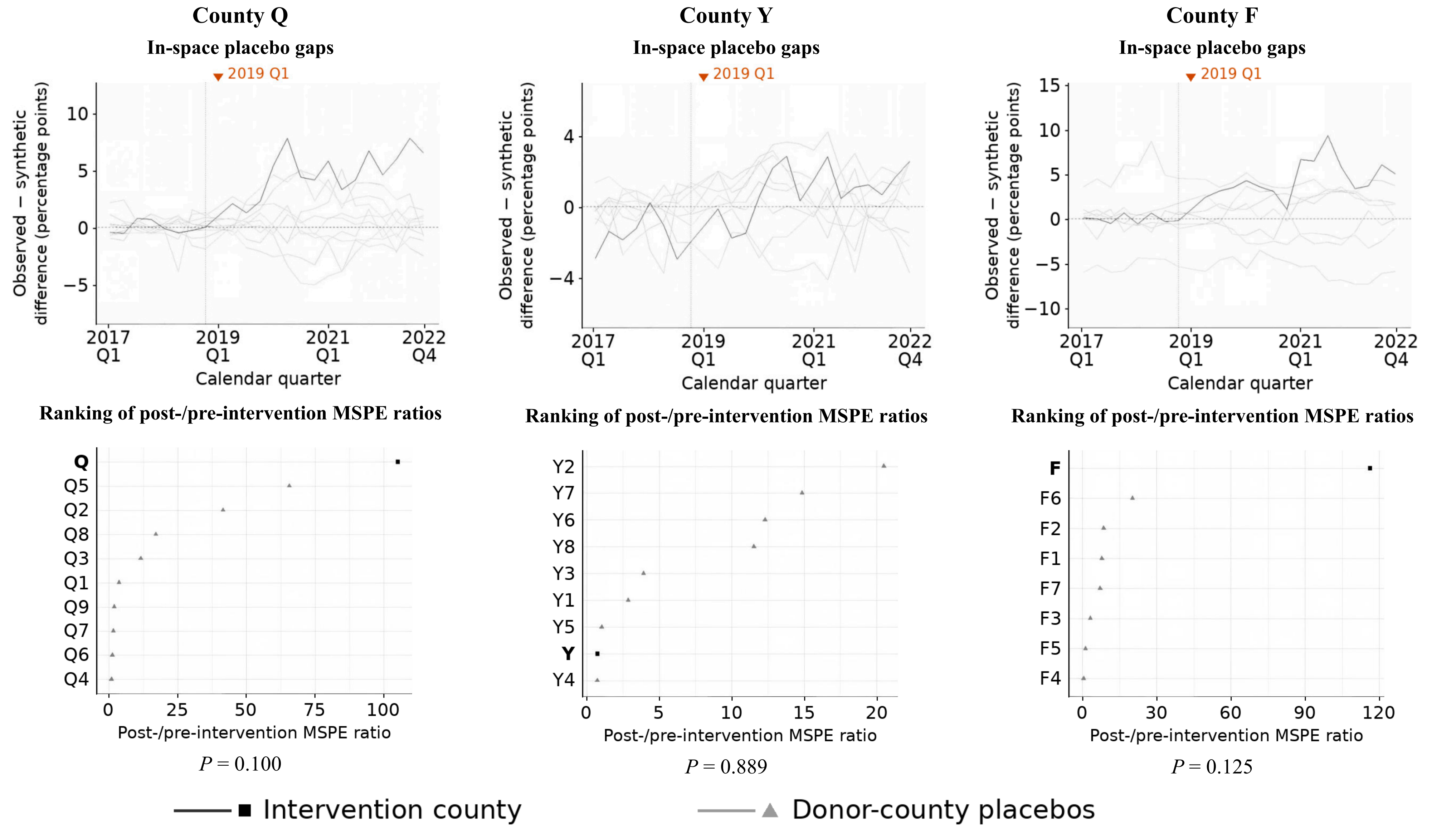

Figure S2. In-space placebo comparisons for the glycemic control rate among patients with T2DM.

Notes. Upper panels show quarterly observed-minus-synthetic differences; lower panels rank post-/pre-intervention MSPE ratios. The triangle marks 2019 Q1, the first intervention quarter; the vertical dotted line marks the end of 2018 Q4. MSPE, mean squared prediction error.

Table S3. SCM diagnostics and estimated county-specific differences

Intervention county	Outcome	Pre-intervention RMSPE	Mean post-intervention difference (2019–2022) (pp)	Final-quarter difference (2022 Q4) (pp)	Post-/pre-intervention RMSPE ratio
County Q	Standardized management rate	0.00158	4.57	7.10	30.50
County Q	Glycemic control rate	0.00498	4.61	6.50	10.25
County Y	Standardized management rate	0.00793	3.96	4.80	5.92
County Y	Glycemic control rate	0.01924	0.83	2.50	0.87
County F	Standardized management rate	0.01441	4.35	6.21	3.08
County F	Glycemic control rate	0.00479	3.81	5.12	10.77

Notes. Differences are expressed in percentage points (pp) and calculated as 100 × (observed proportion − synthetic proportion). RMSPE values are expressed on the 0–1 proportion scale. Pre-intervention RMSPE was calculated over 2017 Q1–2018 Q4; the post-intervention period was 2019 Q1–2022 Q4. Mean differences are averages of the 16 post-intervention quarterly gaps; final-quarter differences refer to 2022 Q4. Differences are rounded to two decimal places. The post-/pre-intervention RMSPE ratio equals post-intervention RMSPE divided by pre-intervention RMSPE and is dimensionless. SCM, synthetic control method; RMSPE, root mean squared prediction error. MSPE, mean squared prediction error. Placebo comparisons in Figures S1 and S2 report MSPE ratios, which are the squares of the corresponding RMSPE ratios.

Table S4. Sensitivity of SCM estimates to maximum-weight donor exclusion

Intervention county	Outcome	Original pre-intervention RMSPE	Original mean post-intervention difference (pp)	Maximum-weight donor excluded	Pre-intervention RMSPE ratio (re-estimated/original)	Re-estimated mean post-intervention difference (pp)	Relative change in mean post-intervention difference (%)
County F	Standardized management rate	0.01441	4.35	County 3	1.07	2.09	-52.05
County F	Glycemic control rate	0.00479	3.81	County 3	1.25	3.09	-18.93
County Q	Standardized management rate	0.00158	4.57	County 9	2.78	2.72	-40.59
County Q	Glycemic control rate	0.00498	4.61	County 4	1.49	5.06	9.75
County Y	Standardized management rate	0.00793	3.96	County 7	1.15	3.48	-12.23
County Y	Glycemic control rate	0.01924	0.83	County 6	1.15	0.29	-65.05

Notes. Each model was re-estimated after excluding its maximum-weight donor. Mean post-intervention differences cover 2019 Q1–2022 Q4; pre-intervention RMSPE covers 2017 Q1–2018 Q4. Differences are expressed in percentage points (pp); RMSPE values are expressed on the 0–1 proportion scale. The pre-intervention RMSPE ratio equals the re-estimated pre-intervention RMSPE divided by the original pre-intervention RMSPE and is dimensionless. Relative change (%) = (re-estimated mean difference − original mean difference) / original mean difference × 100. SCM, synthetic control method; RMSPE, root mean squared prediction error. Differences are rounded to two decimal places; relative changes (%) may not be reproduced exactly from the displayed rounded values.
